# *Drosophila* models of the anti‐inflammatory and anti‐obesity mechanisms of kombucha tea produced by *Camellia sinensis* leaf fermentation

**DOI:** 10.1002/fsn3.4223

**Published:** 2024-05-19

**Authors:** Duy Binh Tran, Nguyen Khoi Nguyen Le, Minh Tue Duong, Kamo Yuna, L. A. Tuan Pham, Q. C. Thanh Nguyen, Yingmanee Tragoolpua, Thida Kaewkod, Kaeko Kamei

**Affiliations:** ^1^ Department of Functional Chemistry Kyoto Institute of Technology Kyoto Japan; ^2^ Department of Surgery, College of Medicine University of Illinois Chicago Illinois USA; ^3^ Department of Molecular Pathology Hanoi Medical University Hanoi Vietnam; ^4^ Department of Chemistry, College of Natural Sciences Cantho University Cantho City Vietnam; ^5^ Department of Biology, Faculty of Science Chiang Mai University Chiang Mai Thailand; ^6^ Natural Extracts and Innovative Products for Alternative Healthcare Research Group, Faculty of Science Chiang Mai University Chiang Mai Thailand; ^7^ Research Center of Deep Technology in Beekeeping and bee Products for Sustainable Development Goals (SMART BEE SDGs), Faculty of Science Chiang Mai University Chiang Mai Thailand

**Keywords:** anti‐inflammation, anti‐obesity, *drosophila* lipid storage droplet‐1, JNK pathway, Kombucha tea

## Abstract

Kombucha tea is a traditional beverage originating from China and has recently gained popularity worldwide. Kombucha tea is produced by the fermentation of tea leaves and is characterized by its beneficial properties and varied chemical content produced during the fermentation process, which includes organic acids, amino acids, vitamins, minerals, and other biologically active compounds. Kombucha tea is often consumed as a health drink to combat obesity and inflammation; however, the bioactive effects of kombucha tea have not been thoroughly researched. In this study, we reveal the underlying mechanisms of the beneficial properties of kombucha tea and how they protect against obesity and inflammation by studying *Drosophila* models. We established an inflammatory *Drosophila* model by knocking down the lipid storage droplet‐1 gene, a human perilipin‐1 ortholog. In this model, dysfunction of lipid storage droplet‐1 induces inflammation by enhancing the infiltration of hemocytes into adipose tissues, increasing reactive oxygen species production, elevating levels of proinflammatory cytokines, and promoting the differentiation of hemocytes into macrophages. These processes are regulated by the c‐Jun N‐terminal Kinase (JNK) pathway. Using this unique *Drosophila* model that mimics mammalian inflammation, we verified the beneficial effects of kombucha tea on reducing tissue inflammation. Our data confirms that kombucha tea effectively improves inflammatory conditions by suppressing the expression of cytokines and proinflammatory responses induced by lipid storage droplet‐1 dysfunction. It was found that kombucha tea consumption alleviated the production of reactive oxygen species and activated the JNK signaling pathway, signifying its potential as an anti‐inflammatory agent against systemic inflammatory responses connected to the JNK pathway. Kombucha tea reduced triglyceride accumulation by increasing the activity of *Brummer* (a lipase), thereby promoting lipolysis in third‐instar larvae. Therefore, kombucha tea could be developed as a novel, functional beverage to protect against obesity and inflammation. Our study also highlights the potential use of this innovative model to evaluate the effects of bioactive compounds derived from natural products.

## Graphical abstract

Kombucha tea effectively promotes lipid degradation through the activation of lipase. It attenuates inflammation in *Drosophila* caused by LSD‐1 dysfunction, which closely resembles mammalian inflammation. Additionally, it inhibits the release of proinflammatory cytokines and reduces ROS production by preventing the translocation of *p*‐JNK from the cytoplasm to the nucleus in intestinal cells.

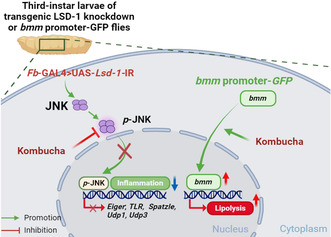

## 1 INTRODUCTION

Kombucha tea is a refreshing beverage that is made by fermenting *Camellia sinensis* (*C. sinensis*) leaves with a mutualistic culture of yeast and acetic acid bacterial cells. It is widely consumed as a traditional drink in China, Thailand, Vietnam, Russia, and Germany (Dufresne & Farnworth, 2000). Kombucha has been consumed and popularized because of its unique flavor, and is said to possess various health benefits, such as stimulating the detoxification of the liver (Jayabalan et al., 2010), improving glandular and gastric functions (Banerjee et al., 2010), reducing arthritis pain (Maheshwari et al., 2014), decreasing body weight and cholesterol concentrations (Yang et al., 2009), and supporting the immune system (Afsharmanesh & Sadaghi, 2013). During the fermentation process, the microorganisms present in yeast and bacterial cells produce potentially favorable substances, including amino acids, vitamins, minerals, organic acids, and biologically active compounds (Kaewkod et al., 2019; Villarreal‐Soto et al., 2018). The production of these natural compounds derived through plant fermentation has been investigated extensively (Twaij & Hasan, 2022), and compounds such as flavonoids and polyphenols are found to have protective properties against many disorders and diseases, including atherosclerosis, neurodegenerative diseases, obesity, and inflammation (Liu, 2004). Several studies on kombucha tea have been conducted, primarily analyzing its antibacterial, antioxidant, hypolipidemic, anticancer, and antidiabetic activities (Chakravorty et al., 2019). However, despite being consumed for a few decades, there is still a lack of comprehensive scientific reports confirming its properties and benefits.

Inflammation is a response to defend the body from harmful stimuli in the form of infections and tissue damage, for example, and to restore normal body functions (Fürst & Zündorf, 2014). Misregulated inflammation, however, causes the misexpression of proinflammatory mediators and, in turn, several diseases, including diabetes, cancer, and cardiovascular diseases (Michaud et al., 2012). In addition, such pathologies are evident by the upregulation of proinflammatory cytokine production and reactive oxygen species (ROS) production, which can cause metabolic dysregulation in adipose tissues and the liver (Scrivo et al., 2011). In mammals, adipocytes regulate lipid homeostasis, control energy storage, and function as endocrine organs to produce various proinflammatory cytokines that can contribute to the synthesis and secernment of inflammatory cytokines, such as tumor necrosis factor‐alpha (TNF‐α), interleukin‐1‐beta (IL‐1β), and interleukin‐6 (IL‐6) (Fantuzzi, 2005). Notably, *Drosophila melanogaster* and mammals share similar lipids and metabolism‐related genes (Heier & Kühnlein, 2018). For example, adipocyte and glycogen energy storage occurs within fat bodies in *Drosophila*, which is analogous to the liver and white adipose of mammals (Canavoso et al., 2001). Lipid droplets (LDs) contain neutral lipids, which consist of sterols and free fatty acids (FFAs) in the form of cholesterol esters and triglycerides, respectively, and they play an essential role in energy homeostasis (Guo et al., 2009). Storing lipid metabolites as LDs helps to reduce the harmful effects of excess FFAs or sterols, which can lead to insulin resistance and inflammation (Hoo et al., 2017; Iyer et al., 2010).

Perilipins are key factors related to the control of lipid mobilization in response to energy provision. (Beller et al., 2010; Bickel et al., 2009) In mammals, lipid metabolism is regulated by perilipins, which can either facilitate or hinder the interaction of lipases, for example, hormone‐sensitive lipase (HSL) and adipose triglyceride lipase (ATGL) with LDs (Beller et al., 2010; Bickel et al., 2009). Perilipins also regulate the activation of ATGL by binding to comparative gene identification 58 (CGI‐58) (Beller et al., 2010). Partial lipodystrophy and type 2 diabetes are frequently tied to genetic variations of perilipins (Bickel et al., 2009; Granneman et al., 2009). In mice, the depletion of perilipin 1 (PLIN1) results in a lean phenotype, accompanied by insulin resistance and glucose intolerance. The depletion of PLIN1 and mutation of the leptin receptor gene can help to prevent obesity, given that PLIN1 deficiency induces an inflammatory response in fat through the dysregulation of lipolysis (Sohn et al., 2018). The fruit fly has genes encoding two perilipins, including *Plin1* (namely lipid storage droplet‐1—*Lsd‐1*) and perilipin 2 (namely lipid storage droplet‐2—*Lsd‐2*) (Miura et al., 2002). *Lsd‐1* resides on the surfaces of LDs of varying sizes, whereas the location of *Lsd‐2* is frequently restricted to smaller LDs (Bi et al., 2012). Moreover, the roles of these genes have been validated in the development of the wing and salivary glands of *Drosophila* (Bi et al., 2012; Binh et al., 2022; Binh, Pham, Men, Dang, & Kamei, 2019; Men, Binh, et al., 2016). LSD‐1 is necessary for the regulation of apoptosis and the activation of the JNK pathway in the thorax development of *Drosophila* (Binh, Pham, Men, & Kamei, 2019). As the JNK pathway is related to inflammation (Roy et al., 2008), we assumed that LSD‐1 had a crucial role in governing inflammation. The hypothesis prompted us to analyze the *Lsd‐1* knockdown‐induced inflammatory responses in *Drosophila*.

This study reveals the underlying mechanisms of the protective effects of kombucha tea against obesity and inflammation using a *Drosophila* model. First, we established an inflammation model using *Lsd‐1* knockdown in the adipose tissues of *Drosophila* and confirmed the phenotype that mimics the inflammatory responses in humans. Second, we observed the anti‐inflammatory and anti‐obesity activities of kombucha tea using the inflammation model and a lipase (Brummer [*bmm*]) expression monitoring model of *Drosophila*, which were established in the present study and in our previous study, respectively. We found that the oral administration of kombucha tea increased lipolysis activation by promoting lipase expression. These results provide valuable information about the potential use of kombucha tea in mitigating obesity and inflammation.

## 2 MATERIALS AND METHODS

### 2.1 The process of preparing kombucha tea using *C. sinensis* leaves

The *C. sinensis* leaves and fermentation culture were supplied by Tea Gallery Group, located in Chiang Mai, Thailand, a company specializing in the production of commercial kombucha tea products. Dried black tea leaves were placed in 500 mL of sterilized water at 1% (*w/v*) and cooked for 15 min. Sucrose was sieved and added to the hot tea at 10% (*w/v*). A consortium culture of acetic acid bacteria and yeast at 7.79–7.81 and 7.53–7.75 log CFU/mL, respectively, was used to prepare 10% (*v/v*) starter broth that was added to the tea and sugar mixture and then further incubated at 25°C for 15 days (Kaewkod et al., 2019). The supernatant was filtrated using No. 1 filtration paper (Advantech, Tokyo, Japan) and stored at 4°C as a stock solution.

### 2.2 Fly stocks

Fly stocks were maintained at 25°C with standard food (Men et al., 2022). All feeding experiments used yellow‐white (*yw*) flies as controls, which are commonly used as a reference in laboratory experiments (Ryuda et al., 2008). RNAi lines carrying UAS‐*GFP*‐IR (#9330) were bought from Bloomington Stock Center (BDSC, IN, USA). To obtain other controls, the female *Fb*‐GAL4 driver was used to cross UAS‐*GFP*‐IR and *yw* flies separately. UAS‐*Lsd‐1*‐IR_119‐125_ (#60530, BDSC) (Ni et al., 2009) and UAS‐*Lsd‐1‐*IR_170‐220_ (#106891; Vienna *Drosophila* Resource Center—VDRC, Vienna, Austria) (Heigwer et al., 2018) carry an inverted repeat (IR) of *Lsd‐1* downstream of the UAS sequence (targeting the region of amino acids from 119 to 125 and 170 to 220, respectively). The RNAi target sequences were designed to avoid any off‐target effects (online ds Check software of VDRC, http://dscheck.rnai.jp).

Flies carrying the *bmm* promoter‐GFP transgene, which were generated in our laboratory, were created by introducing GFP as a downstream reporter of the *bmm* promoter (Men, Thanh, et al., 2016). The *Fb*‐GAL4 driver line (+; *Fb*‐GAL4; +) was provided by Ronald P. Kühnlein of the Max Planck Institute for Biophysical Chemistry (Göttingen, Germany) (Gronke et al., 2003).

### 2.3 Feeding assay

For mating, 10 male and 30 female flies were kept at 25°C for 24 h on a diet of standard food, then held for 2 h to lay eggs in order to collect a synchronized age of larvae (Binh, LAP, Nishihara, Thanh Men, & Kamei, 2019). Larvae were moved to two new cages, both containing the Formula 4–24 Instant Drosophila medium (#173200, Carolina Biological, NC, USA), one with kombucha tea, and one without.

Second‐instar larvae were fed a standard diet containing 2.5% (w/v) Brilliant Blue R (Sigma‐Aldrich, MA, USA), along with kombucha tea (6%, w/v), for 24 h to assess their feeding rate compared to the untreated control group. The compounds rutin and kaempferol were dissolved with distilled water (DW) and adjusted to concentrations of 400 μM and 40 μM, respectively, before feeding. Ten larvae were crushed in 200 μL DW, and 800 μL DW was added. The supernatant was collected after filtration using filter paper. The absorbance of Brilliant Blue R was measured at 630 nm using a microplate reader (SH‐1200; Corona Electric, Japan).

### 2.4 Hemocyte observation

The protocol established in a previous study (Petraki et al., 2015) was used to evaluate the number of circulating and resident hemocytes. Briefly, third‐instar larvae were cleaned with DW by soft brushing, then placed in 70% ethanol and rinsed with phosphate‐buffered saline (PBS). Each larva was placed on a Hendley‐Essex 12 multispot slide (Loughton, United Kingdom) containing 30 μL of Schneider's medium (Thermo Fisher Scientific, MA, USA) to count the circulating hemocytes. The anterior and posterior ends of the larvae were excised using two dissecting scissors. The hemocytes were allowed to bleed for 10 sec without any pressure or physical agitation. The larvae were then gently transferred to the next well after the circulating hemocytes were collected to count the resident hemocytes. The lymph glands of the larvae were scraped to separate the hemocytes, which were then counted using a hemocytometer (Lauda‐Königshofen, Germany). Images were obtained under a microscope (Olympus, Tokyo, Japan).

### 2.5 Immunostaining

Adipose tissues, located in the intestines, were incubated with 4% paraformaldehyde (FFA) in PBS for 10 min and stained with antibodies (Binh et al., 2022; Kaewkod et al., 2019). The sample was probed with two specific primary antibodies, mouse anti‐*p‐*JNK G9 antibody (dilution at 1:250, #9255, Cell Signaling Technology—CST, MA, USA) and rabbit anti‐LSD‐1 (diluted at 1:3000, Sigma‐Aldrich), and then incubated overnight at 4°C. Next, the primary antibodies were washed once with PBS before incubation with a specific secondary antibody, anti‐mouse IgG Fluor™ Alexa 488 or 594 (Molecular Probes, Invitrogen, MA, USA), at 1:800 dilution, and was then incubated at 25°C for 1 h. For nuclear staining, DAPI (Molecular Probes, Eugene, OR, USA) was used.

The samples were observed under a confocal microscope (Olympus FLUOVIEW FV10i, Tokyo, Japan) at equal magnification and intensity after being raised in Vectashield mounting medium (Vector Laboratories, Tokyo, Japan). The number of cells with *p*‐JNK translocated from the cytoplasm into the nuclei was counted, based on the signal of *p*‐JNK‐positive cells overlapping with the signal of DAPI‐positive cells at the same magnification using MetaMorph v7.7.7.0 (Molecular Devices, USA).

### 2.6 Quantification of triglyceride (TG)

The TG content was analyzed using the colorimetric sulfo‐phospho‐vanillin (SPV) method (Men, Thanh, et al., 2016). Third‐instar larvae (10 mg) were homogenized in 2% Na_2_SO_4_ (*w/v*), then chloroform and methanol were added 1:1, (*v/v*). The supernatant was obtained through centrifugation (1000 × *g*, 1 min) and mixed with DW. For lipid measurements, the chloroform layer was dried (90°C, 10 min) and 10 μL of 98% sulfuric acid (Sigma‐Aldrich) was added, then incubated at 90°C for 10 min. After the mixture was cooled to 25°C, 990 μL of SPV reagent was added to the solution, and the color development was measured at 530 nm using a microplate reader. The TG content is expressed relative to the content of oleic acid (Sigma‐Aldrich), and was used as a standard.

### 2.7 ROS detection

Intestines from the middle stage of third‐instar larvae were collected and immediately incubated with 10 μM of CM‐H_2_DCFDA (Molecular Probes, Invitrogen, USA) in PBS for 5 min (Binh, Pham, Men, & Kamei, 2019). Samples were examined under a confocal microscope (Olympus FLUOVIEW FV10i) at equal magnification and intensity after being raised in Vectashield mounting medium. The intensity of fluorescence was analyzed using MetaMorph v7.7.7.0 (Pham et al., 2020).

### 2.8 Reverse transcription‐quantitative polymerase chain reaction (RT‐qPCR)

The whole body of a middle‐stage third‐instar larva was crushed to extract the total RNA. This was done using standard Qiazol reagent (Qiagen, Germany), and was purified using the Qiagen RNeasy kit (Qiagen). The SimpliAmp thermal cycler (Life Technologies, Singapore) was used to synthesize the cDNA. The master mix of the reaction included 100 ng of cDNA, FastStart Essential DNA Green Master Mix, and 100 nM of forward primer and reverse primer. RT‐qPCR was performed using LightCycler® 96 (Roche). The sequences of the gene‐specific primers for *rp49* acted as an internal control, and the other genes are presented in Table 1. The cycle threshold (Ct) values of the genes were analyzed by relative expression and normalized with the expression of the regulator gene (*rp49*).

**TABLE 1:** Oligonucleotides used for RT‐qPCR.

Genes	Sequences of PCR primers (5′‐3′)
*rp49*	Forward: ACCAGCTTCAAGATGACCATCC Reverse: CTTGTTCGATCCGTAACCGATG
*Lsd‐1*	Forward: TCTGGAGCAGTTGATTGTGC Reverse: AATATGGTGGGCAACCTCTG
*bmm*	Forward: GGCAATGGGAACAACTGAAC Reverse: TTGATCGGGCAATTTGATGATCC
*hsl*	Forward: GCAGGAACAACTGATGGAAC Reverse: GCAACGGGCAATTTGATGATCC
*Eiger*	Forward: GCCATTCTCGCACTAACGAT Reverse: TTATCGACGACTCGCTTCAG
*TLR*	Forward: GTAACCAAACGGGGAGTTGA Reverse: AACTTGGGCAACCTTGTGAC
*Spatzle*	Forward: AGTCCTGCTGCTGCTGTTC Reverse: CTCGTGTCGCTTCGTTAATTC
*Udp1*	Forward: CAAGCGGGTAAGGAGTCG Reverse: TGCTGCTGCTGTAGCAACTT
*Udp3*	Forward: CCCAGCCAACGATTTTTATG Reverse: TGTTACCGCTCCGGCTAC

### 2.9 Statistical analysis

Experiments were performed at least three times. The statistical analysis and figure preparation were conducted in MS Excel 2010 (Microsoft Corp., Redmond, WA, USA) and GraphPad Prism v9.2.0 (GraphPad Software Inc., San Diego, CA, USA). The results are expressed as the mean ± standard deviation (SD). Statistically significant differences in means were determined through a one‐way analysis of variance followed by Tukey's multiple tests. The statistical significance was set at *p* < .05.

## 3 RESULTS

### 3.1 LSD‐1 dysfunction induces inflammation in the adipose tissues of drosophila larvae

**FIGURE 1:**
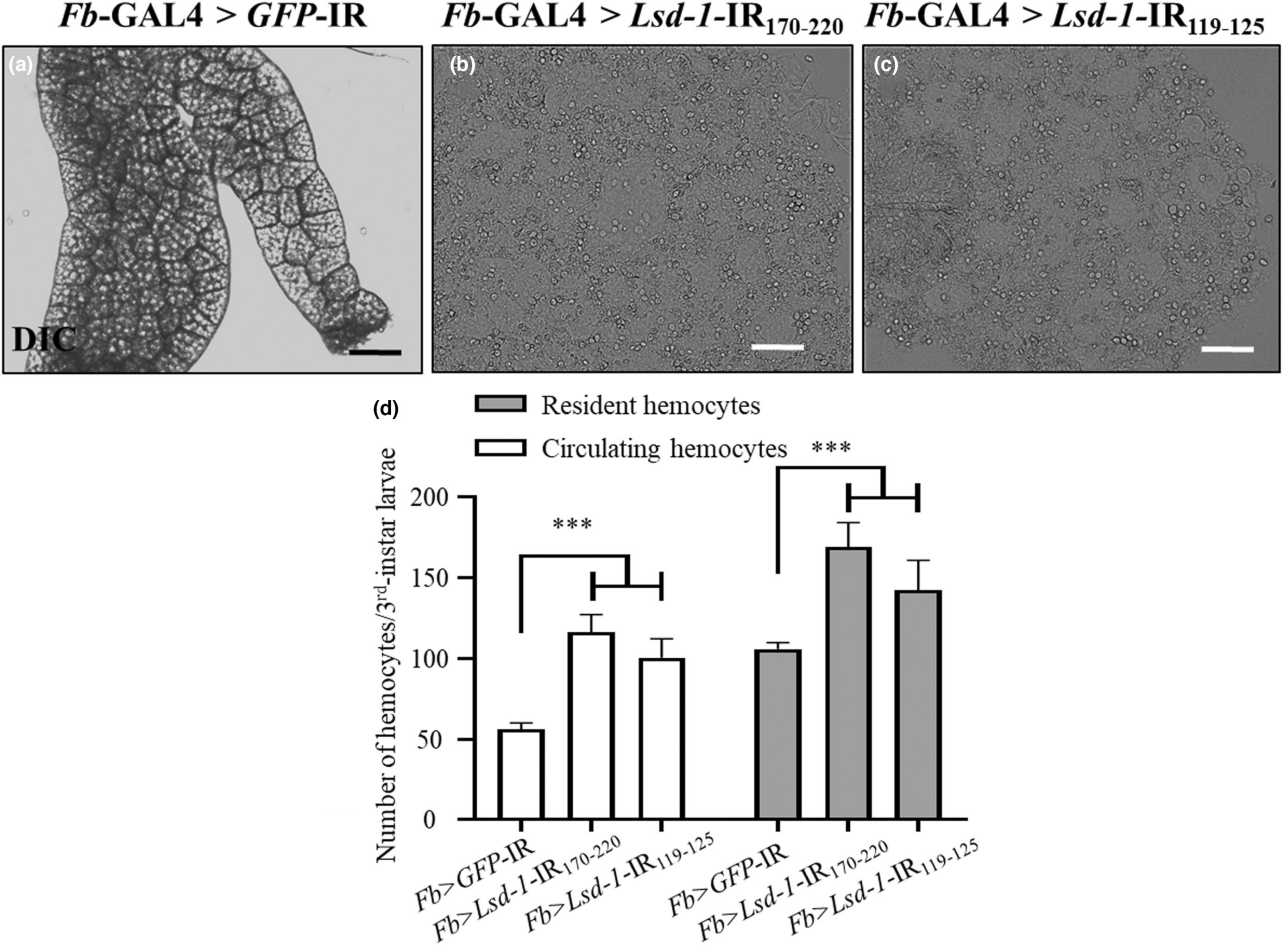
LSD‐1 dysfunction in adipocytes induces fat cell dissociation and hemocyte infiltration. Differential interference contrast (DIC) images of fat bodies (a–c) at the middle stage of third‐instar larvae are shown. Control flies were generated by mating *Fb*‐GAL4 driver flies with UAS‐*ds*GFP RNAi flies (a). *Fb* > *Lsd‐1*‐IR is characterized by fat cell dissociation and hemocyte accumulation in the fat bodies (b) and (c). The count of resident and circulating hemocytes was assessed during the early stage of third‐instar larvae in both the control and *Lsd‐1* KD (*Fb > Lsd‐1*‐IR_170‐220_ and *Fb > Lsd‐1*‐IR_119‐125_) (d, *n* = 4, 50 larvae per group). Error bars represent the mean ± SD. Significant differences were analyzed statistically using one‐way analysis of variance, followed by Tukey's multiple comparison tests. Scale bar: 50 μm; ***, *p <* .001. Genotypes: (a) (+; *Fb*‐GAL4/+; UAS‐*GFP*‐IR/+), (b) (+; *Fb*‐GAL4/UAS‐*Lsd‐1*‐IR_170‐220_; +), (c) (+; *Fb*‐GAL4/UAS‐*Lsd‐1*‐IR_119‐125_; +).

To explore the function of LSD‐1 in *Drosophila* inflammation, the expression of LSD‐1 was depleted in various tissues via the expression of *Lsd‐1* dsRNA using the GAL4/UAS system (Brand & Perrimon, 1993). Two different *Lsd‐1* RNAi strains were crossed with *Fb*‐GAL4 to knock down *Lsd‐1* expression in adipose tissues, intestines, and salivary glands, thereby excluding the possibility of off‐target effects (Rynes et al., 2012; Schmid et al., 2014). Our findings were consistent with those of a previous study, indicating that the third‐instar larvae of both knockdown strains exhibited a normal‐looking phenotype (Beller et al., 2010). However, the fat bodies of the middle stage (98 h after embryos hatch [AEH]) of third‐instar larvae of both *Lsd‐1* knockdown (*Lsd‐1* KD) flies were characterized by fat cell dissociation and hemocyte infiltration, indicating inflammatory responses (Figure 1a–c). Immunostaining and the RT‐qPCR results showed that the protein expression and mRNA levels of LSD‐1 decreased significantly in the midgut of the KD flies, *Fb* > *Lsd‐1*‐IR (Figure S1).

To confirm the inflammatory response, the number of circulating and resident hemocytes was tallied in the early stage (larvae between 82 and 84 h old AEH) of both the knocked‐down and the control third‐instar larvae (Figure 1d). The results showed that the numbers of both hemocytes of the two KD flies, namely, *Fb >* UAS‐*Lsd‐1*‐IR_170‐220_ and *Fb >* UAS‐*Lsd‐1*‐IR_119‐125_, were significantly higher than those of the control flies (*Fb > GFP*‐IR). These results indicate that *Lsd‐1* knockdown induced inflammation in *Drosophila* third‐instar larvae.

### 3.2 Lsd‐1 knockdown causes inflammation dependent on the JNK pathway

**FIGURE 2:**
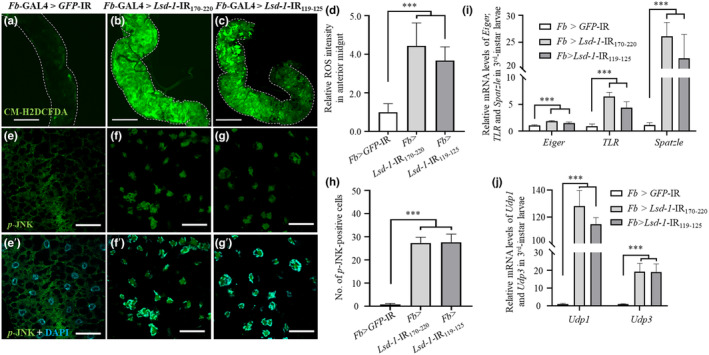
The suppression of *Lsd‐1* leads to the production of ROS and the translocation of *p*‐JNK into the nucleus in the anterior midgut of third‐instar larvae. ROS in the anterior midgut of the third‐instar larvae of the control (*Fb‐GAL4 > GFP*‐IR, a) and *Lsd‐1* KD flies (b) and (c) were detected using CM‐H2DCFDA. The controls showed undetectable ROS signals in the anterior midgut cells, whereas *Lsd‐1* KD flies (*Fb > Lsd‐1*‐IR_170‐220_) exhibited distinct ROS signals. The dotted line in (a–c) indicates the anterior midgut area. The relative ROS intensity in the anterior midgut is shown (d, *n* = 30 for each genotype). The anterior midgut tissues of the third‐instar larvae of the control (e) and *Lsd‐1* KD (f and g) flies were subjected to staining with a rabbit anti‐*p*‐JNK antibody. Merged images of rabbit anti‐*p*‐JNK and DAPI signals (e′, f′, and g′) are shown. *Lsd‐1* KD (e–g) flies showed translocated *p*‐JNK signals in the nuclei. The number of *p*‐JNK‐positive cells is shown (H, *n* = 30 for each genotype). The expression levels of *Eiger*, *TLR*, *Spatzle*, *Udp1*, and *Udp3* mRNA in the entire third‐instar larvae of *Lsd‐1* KD flies are presented relative to those of the control flies (i, j, *n* = 4). The error bars depict the mean ± SD. Significant differences were assessed through a one‐way analysis of variance, followed by Tukey's multiple comparison tests. Scale bar: 50 μm (a–c) and 200 μm (e–g and e′–g′); ***, *p <* .001. Genotypes: (a, e, e′) (+; *Fb*‐GAL4/+; UAS‐*GFP*‐IR/+), (B, F, F′) (+; *Fb*‐GAL4/UAS‐ *Lsd‐1*‐IR_170‐220_; +), and (c, g, g′) (+; *Fb*‐GAL4/UAS‐ *Lsd‐1*‐IR_119‐125_; +).

To determine whether *Lsd‐1* knockdown resulted in an increase in ROS production, we examined the possibility of ROS acting as proinflammatory mediators that play a crucial role in inflammation. In the present experiment, we used the intestine because the dissociated adipose tissues of the knockdown flies were impossible to collect. We used CM‐H2DCFDA to detect and evaluate intracellular ROS (Martinez‐Botas et al., 2000). The results showed that the ROS signals in the intestines of *Lsd‐1* KD flies were stronger than those in the control flies (Figure 2a–d).

LSD‐1 is vital for controlling the JNK signaling pathway (Binh, Pham, Men, & Kamei, 2019). Inflammation is associated with JNK activation (Kyriakis et al., 1994); therefore, we analyzed the activation of JNK in the intestines of *Lsd*‐*1* KD by immunostaining with a *p*‐JNK antibody. Figure 2 shows that the *p*‐JNK signal was translocated from the cytoplasm into the nuclei of the intestinal cells of the middle‐stage third‐instar larvae of knockdown flies (Figure 2e–h).

Next, we used RT‐qPCR to examine the expression levels of several key genes involved in the inflammatory response in third‐instar larvae. Eiger is the *Drosophila* homolog of TNF, while Spatzle plays a role similar to its mammalian counterpart in important proinflammatory responses (Paddibhatla et al., 2010; Vidal, 2010). Toll‐like receptors (TLRs) have been recognized as crucial receptors that initiate inflammatory responses (Imler & Hoffmann, 2002). Upd1 and Upd3 are IL‐6‐like cytokines in *Drosophila* (Binh, LAP, Nishihara, Thanh Men, & Kamei, 2019). As anticipated, the mRNA expression of the inflammatory genes was found to be upregulated in the middle stage of the third‐instar larvae of *Lsd‐1* KD flies, compared to the expression in the control flies (Figure 2i,j). The results imply that LSD‐1 dysfunction activates the JNK signaling pathway, thereby inducing an inflammatory response. Therefore, we successfully established an inflammation fly model through *Lsd‐1* knockdown.

### 3.3 Kombucha tea intake reduces the TG content of the third‐instar larvae of *Drosophila*

**FIGURE 3:**
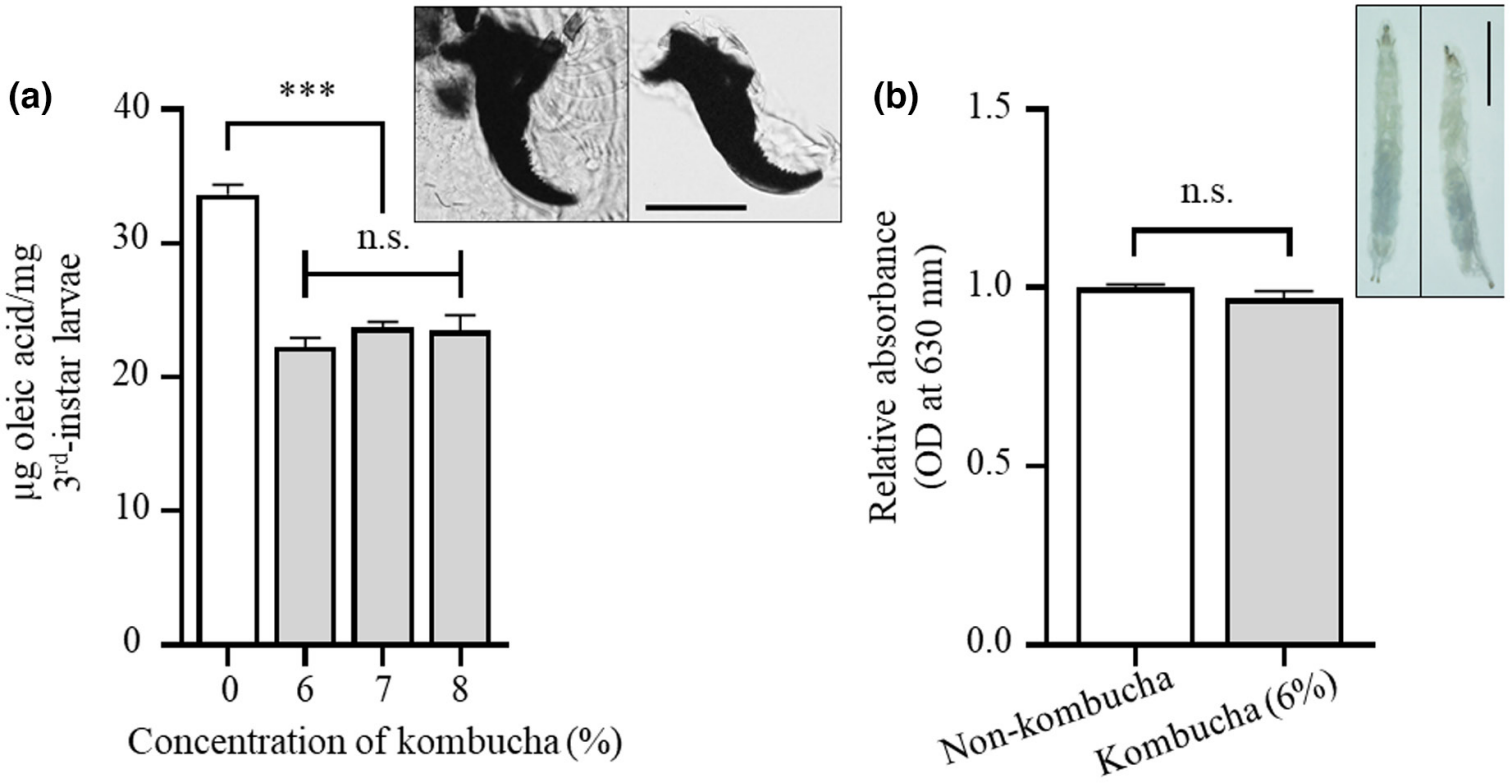
Kombucha tea intake suppresses TG accumulation in third‐instar *Drosophila* larvae. TG content in whole larvae of the *yw* strain fed with the instant medium Formula 4–24 containing different concentrations of kombucha tea was analyzed (a). The upper right panel in Figure A shows the same mouth‐hook morphology with multiple teeth of third‐instar larvae of *yw* flies: left, control diet (non‐kombucha)‐fed larvae; right, kombucha (6%, *w/v*) diet‐fed larvae. The relative absorbance at 630 nm of homogenate of third‐instar larvae fed with standard food containing 2.5% Brilliant Blue R or standard food containing 6% kombucha tea and 2.5% Brilliant Blue R is shown (b). The upper right panel in Figure (b) shows the photographs of the third‐instar larvae of the *yw* strain: left, control‐diet (non‐kombucha)‐fed larvae; right, kombucha (6%, *w/v*) diet‐fed larvae. Error bars represent mean ± SD. Significant differences were assessed through one‐way analysis of variance, followed by Tukey's multiple comparison tests and student's *t*‐test. Scale bar: 50 μm (a), 1 mm (b); n.s., not significant; ***, *p <* .001.

We evaluated whether kombucha tea affected TG accumulation in *Drosophila*. Thirty male flies and 10 female flies of the *yw* strain were crossed and held to lay eggs on a standard food diet. The hatched progenies were collected and continuously cultured on instant medium Formula 4–24 containing various concentrations of kombucha tea (0%, 6%, 7%, and 8% *w/v*). Whole bodies in the middle stages of third‐instar larvae were assayed for their TG content, which is expressed as oleic acid content. Our results demonstrate that kombucha tea intake reduced the TG content in third‐instar larvae but did not significantly affect the larvae cultured with a diet containing 6%–8% kombucha tea (Figure 3a), indicating that kombucha tea might have an influence on the early to middle stages of third‐instar larvae. The upper right panel in Figure 3a shows the same mouth‐hook morphology with multiple teeth of third‐instar larvae in both the control and treated groups, indicating that larval development was not affected by ingesting kombucha tea. A feeding assay was performed to confirm whether food containing kombucha tea was consumed by *Drosophila* larvae. Kombucha tea intake was indirectly estimated based on the absorbance of Brilliant Blue R in standard food at 630 nm. The quantity of food with and without kombucha tea consumed by the larvae was not significantly different (Figure 3b). Therefore, kombucha tea could potentially decrease the accumulation of TG in third‐instar larvae.

### 3.4 Kombucha tea intake affects the lipid metabolism of third‐instar larvae

**FIGURE 4:**
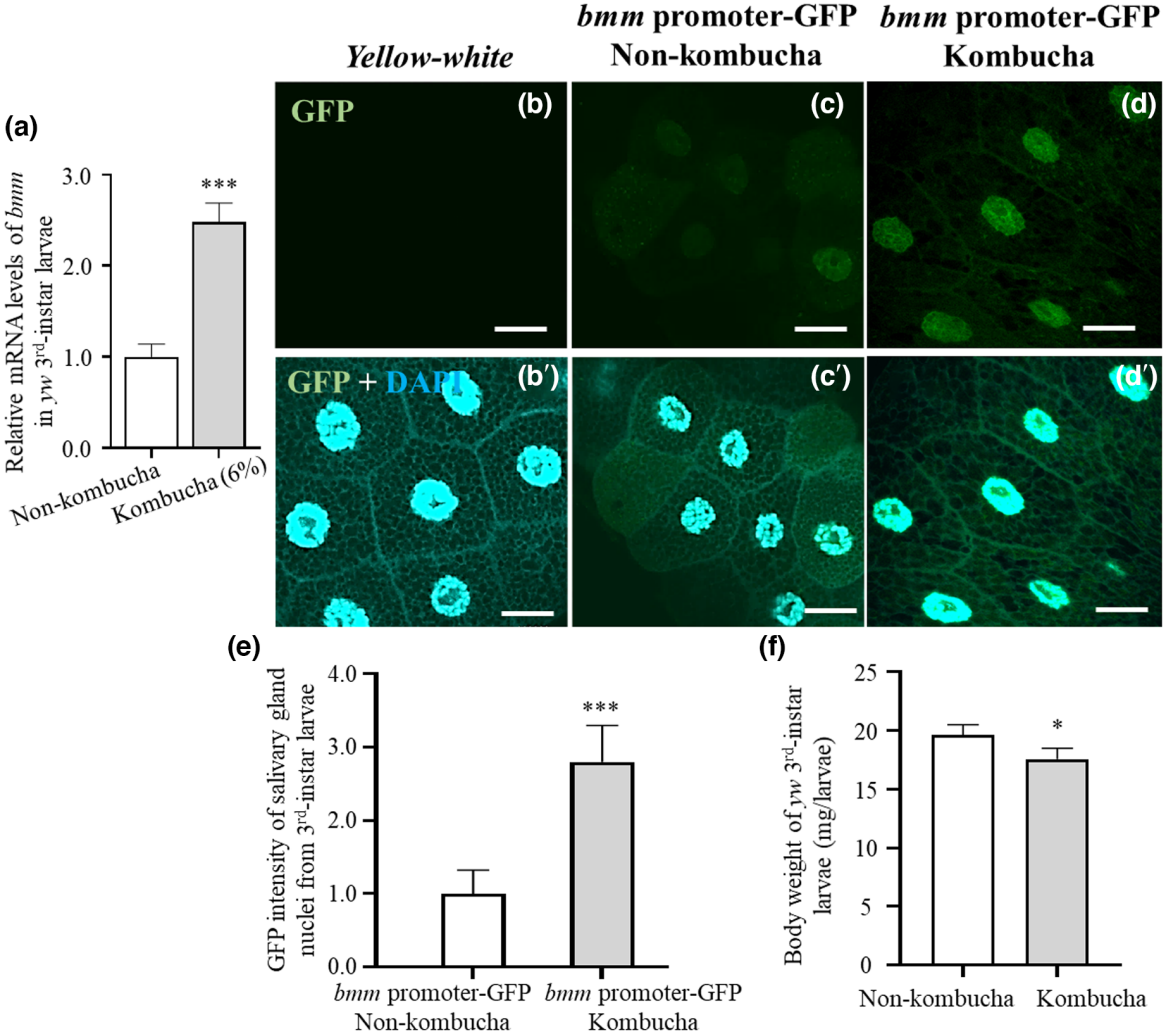
Kombucha tea intake affects lipid metabolism. The relative *bmm* levels in the middle stage of the entire bodies of *yw* third‐instar larvae were analyzed using RT‐qPCR (a, *n* = 4). The *bmm* promoter‐GFP flies were fed with instant food containing or not containing kombucha tea (6%, *w*/*v*), and the third‐instar larvae's salivary glands were analyzed under a confocal microscope (b–d and b′–d′). The images show GFP expression under *bmm* promoter regulation (b–d). The merged images of the GFP and DAPI are displayed (b′–c′). The intensity of GFP signal in the nuclei of the larval salivary glands of transgenic flies of the kombucha tea and control diet (non‐kombucha tea)‐fed groups was analyzed (e, *n* = 30 salivary glands). The whole body weight of *yw* third‐instar larvae in the kombucha tea‐fed and non‐kombucha tea groups is shown (f, *n* = 30, 50 larvae for each phenotype). Error bars depict the mean ± SD. Significant differences were assessed through the Student's *t*‐test. Scale bar: 50 μm; ***, *p <* .001. Genotypes: (b, b′) +; +; +, (c, c′ and d, d′) +; +; *bmm* promoter‐*GFP*/+.

We hypothesized that the reduced TG accumulation in the third‐instar larvae of *yw* flies by kombucha tea intake might be associated with the disturbance of lipid metabolism. First, we analyzed the mRNA expression of *bmm* in the middle stage of *yw* third‐instar larvae using RT‐qPCR, which is a homolog of ATGL in mammals. The results illustrated that the *bmm* expression in the kombucha tea‐fed group (Formula 4–24 containing kombucha tea 6%, *w/v*) was elevated, compared to that in the control group (Formula 4–24 without kombucha tea; Figure 4a). Next, we assessed the *bmm* expression using a reporter assay with *bmm* promoter‐GFP flies, which was established by introducing *GFP* as a downstream reporter of the *bmm* promoter. This fly model can be used for screening potential anti‐obesity food and drug candidates (Men, Thanh, et al., 2016). Thirty male flies and 10 virgin female flies of the *bmm* promotor‐GFP fly strain were crossed, and eggs were laid after a diet of Formula 4–24 with kombucha tea (6%, *w/v*). The same number of flies were crossed and bred on a diet of Formula 4–24 without kombucha tea. The intensity of the GFP signal was measured in the salivary gland cells, which contained giant polytene chromosomes in the third‐instar larvae of the progenies. The results showed that the kombucha tea‐fed group had significantly higher *bmm* expression (Figure 4b–e), suggesting that the TG content in larvae had decreased, possibly because of enhanced lipolysis. In addition, the weight of the whole bodies of *yw* third‐instar larvae decreased slightly when fed with kombucha tea (Figure 4f).

### 3.5 Dietary kombucha tea reduces inflammation induced by LSD‐1 dysfunction

Our results indicate that dietary kombucha tea can actively help prevent obesity. Our previous study confirmed that kombucha tea possesses antioxidant properties (Kaewkod et al., 2019), therefore, it's possible that it also possesses anti‐inflammatory properties. To confirm this hypothesis, we evaluated the anti‐inflammatory activities of kombucha tea using the *Lsd‐1* KD‐induced inflammatory *Drosophila* model.

**FIGURE 5:**
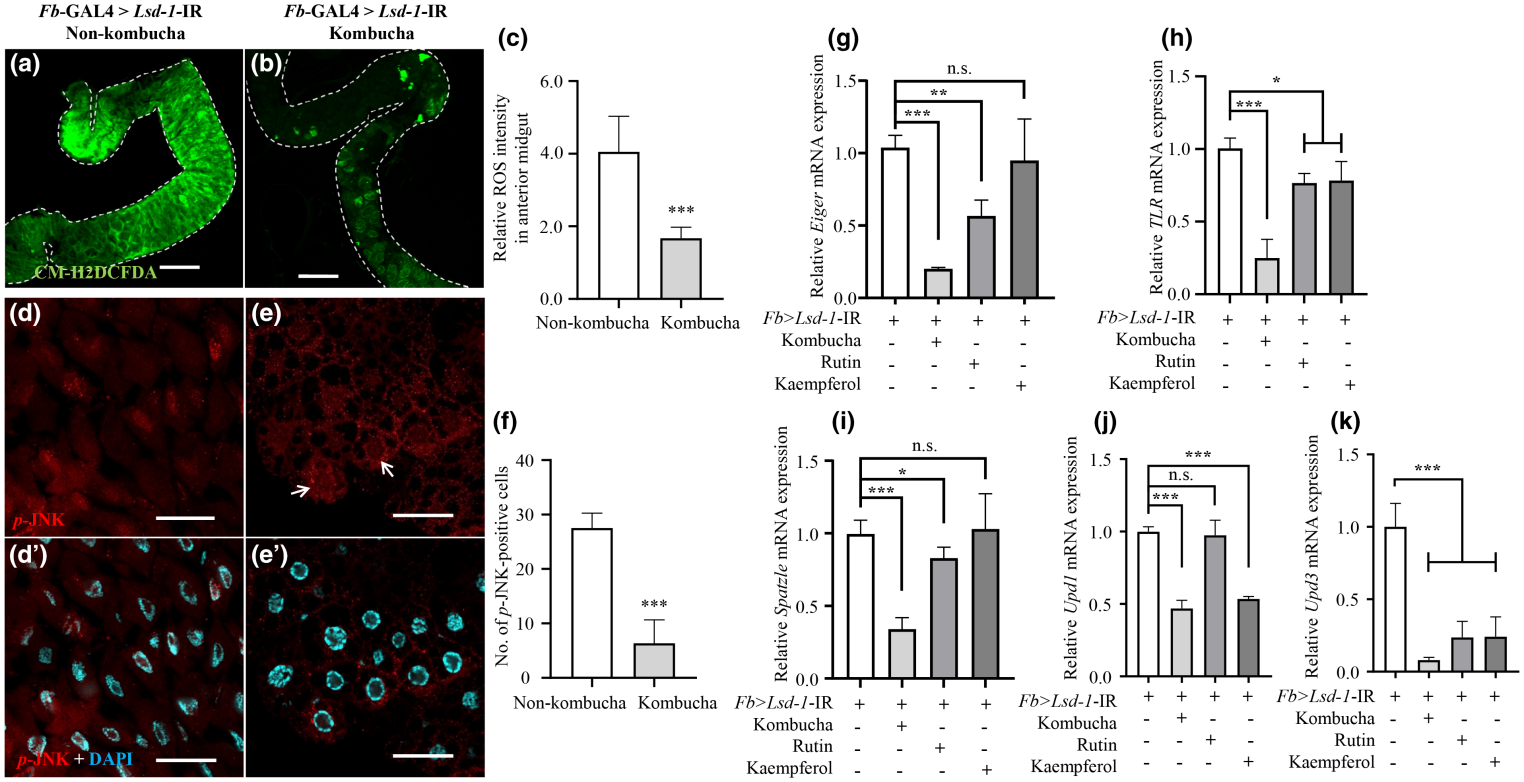
Kombucha tea intake suppresses inflammatory responses induced by LSD‐1 dysfunction. *Lsd‐1* KD flies were fed with instant food without kombucha tea (negative control), with kombucha tea, or with rutin or kaempferol (positive control). ROS in the anterior midgut of the third‐instar larvae of *Lsd‐1* KD, which were fed instant food without kombucha tea (a) or with (b) kombucha tea (6%, *w*/*v*), was detected using CM‐H2DCFDA. The relative ROS intensity in the anterior midgut is shown (c, *n* = 36 for each genotype). The anterior midgut tissues of the third‐instar larvae were also subjected to staining with rabbit anti‐*p*‐JNK antibody (d, e). (d′) and (e') showed the merged images of rabbit anti‐*p*‐JNK and DAPI signals. The number of *p*‐JNK‐positive cells is shown (f, *n* = 36 for each genotype). The dotted line implies the anterior midgut area. White arrows indicate the *p*‐JNK‐positive signal of the nuclei of the intestine, which was suppressed by the oral ingestion of kombucha tea. The relative mRNA expression of genes associated with the inflammatory responses of the whole third‐instar larvae was analyzed using RT‐qPCR (g, *Eiger*; h, *TLR*; i, *Spatzle*; j, *Udp1*; k, *Udp3*; *n* = 4 for each diet group). Error bars represent mean ± SD. Significant differences were assessed through one‐way analysis of variance, followed by Tukey's multiple comparison tests and Student's *t*‐test. Scale bar: 50 μm (a, b) and 200 μm (d–e and d′–e′); n.s.: not significant; *p < .05; **p < .01; ***p < .001. Genotypes: +; *Fb*‐GAL4/UAS‐ *Lsd‐1*‐IR_170‐220_; +.

Thirty male (*Fb*‐GAL4) flies and 10 virgin female (UAS‐*Lsd‐1*‐IR) flies were placed to lay eggs on instant blue medium both with and without 6% (*w/v*) kombucha tea (the kombucha‐fed group and the negative control group, respectively). Rutin (400 μM; Sigma‐Aldrich) and kaempferol (40 μM; Sigma‐Aldrich) were used as positive controls. We examined the effect of the kombucha tea diet on ROS production and JNK phosphorylation in the anterior midgut. Histological staining demonstrated that the ROS signals and *p*‐JNK levels in the kombucha‐fed group were reduced compared to those in the negative control group (Figure 5a–f). The experiments showed that the third‐instar larvae of the kombucha‐fed group and the positive control group had significantly suppressed expressions of the key genes involved in *Drosophila* inflammatory responses (Figure 5g–k). The results indicate that kombucha tea suppresses inflammatory responses by inhibiting the JNK pathway.

## 4 DISCUSSION

Kombucha tea, a common fermented tea beverage, is made by fermenting tea leaves. During fermentation, a plethora of beneficial substances are produced by the symbiotic microbiota and yeast (Jayabalan et al., 2014). According to Kombucha Brewers International (KBI), a trade association for commercial kombucha brewers, over 90% of bottled kombuchas on tap and on store shelves around the world are represented by KBI members. This includes regions in Europe, Asia Pacific, Latin America, and North America (Kim & Adhikari, 2020). Current preliminary studies on kombucha tea focus on its antioxidant, antibacterial, anticancer, and anti‐diabetic properties (Kaewkod et al., 2019; Xu et al., 2022). However, the other favorable effects of kombucha on inflammation mechanisms and obesity, especially in vivo studies, are poorly defined. In this study, we have explored the anti‐obesity and anti‐inflammatory activities of kombucha tea prepared from black tea on *Drosophila*, which has proven to be an excellent model to closely replicate humans. First, we generated an inflammation model through LSD‐1 depletion in adipose tissues, intestines, and salivary glands. Our results indicated that *Lsd‐1* knockdown in adipose tissues induced fat body dissociation and hemocyte infiltration (in macrophage‐like cells) as inflammatory responses. The expression of proinflammatory factors, including *Eiger*, *Spatzle*, *TLRs*, *Upd1*, and *Upd3*, was highly upregulated in *Lsd‐1* KD flies. We observed that ROS production in the intestines of *Lsd‐1* KD flies increased, suggesting that oxidative stress might be responsible for the enhanced *p*‐JNK activation. Intriguingly, Wang et al. described that the depletion of LSD‐1 in the adipocyte suppressed ROS production (Wang et al., 2021), while our previous publication reported that lower LSD‐1 expression in non‐adipocytes (wing pouch and thorax area) increased ROS production (Binh, Pham, Men, & Kamei, 2019; Men, Binh, et al., 2016). Considering the previous reports and our current results, the specific LSD‐1 function seems to be associated with its expression in each tissue. *Drosophila* and humans share similar molecules related to lipid mechanisms, and hemocytes are functionally equivalent to macrophages. Therefore, this model could be used as an inflammation model for humans to assess the effects of kombucha tea and other products on inflammation.

We demonstrated that consuming kombucha tea made from black tea reduced TG accumulation in the third‐instar larvae of *Drosophila*; however, food containing kombucha tea did not affect the food intake levels of *Drosophila* larvae. The results imply that kombucha tea might influence lipid metabolism. The results of RT‐qPCR analysis indicated a significant rise in the mRNA levels of *bmm* in the third‐instar larvae of the group fed with kombucha tea. These results are supported by the reporter assay of the *bmm* promoter in *Drosophila* models. Therefore, it is noted that kombucha tea effectively promoted lipolysis and suppressed lipogenesis in third‐instar larvae by inducing brummer activation. Our previous study verified that kombucha made from black tea contains high antioxidant activity, phenolic compounds, and several organic acids, including gluconic acid, glucuronic acid, ᴅ‐saccharic acid 1,4‐lactone (DSL), acetic acid, succinic acid, and ascorbic acid. (Kaewkod et al., 2019) The function of kombucha tea may be related to the presence of these organic acids. Specifically, acetic acid may inhibit lipogenesis and cholesterogenesis in the liver, which is a metabolic tissue with physiological functions similar to those in *Drosophila* fat bodies (Kapp & Sumner, 2019; Liu et al., 2009). The notable benefits are attributed to the natural components in kombucha tea, such as glucuronic acid and DSL, recognized for their reported capacity to inhibit hyperlipidemia and reduce the occurrence of cardiovascular diseases (Costa et al., 2021). Furthermore, phenolic compounds possess the ability to regulate the gut microbiota and reduce oxidative stress, providing effects that counteract obesity, diabetes, and hypercholesterolemia (Beetch et al., 2020; Gowd et al., 2019).

In the present study, food containing kombucha from black tea suppressed proinflammatory responses, reduced ROS production, and prevented *p*‐JNK translocation induced by LSD‐1 dysfunction, all with significant results compared with the negative control. Moreover, the active flavonoid compounds of kaempferol and rutin found in black tea (Vázquez‐Cabral et al., 2017) also showed inhibitory effects on ROS and inflammation. Our previous study revealed that the concentrations of gluconic and acetic acids are the highest among the organic acids (Kaewkod et al., 2019). Organic acids exhibit various benefits, including the deceleration of gastric emptying, the inhibition of disaccharidase activity, and detoxification of the liver (Kapp & Sumner, 2019; Zubaidah et al., 2019). The antioxidants present in kombucha tea demonstrate the ability to neutralize ROS and mitigate the oxidation of cellular molecules, thereby alleviating oxidative stress (Gilgun‐Sherki et al., 2001; Kaewkod et al., 2019). The polyphenols found in kombucha tea may play a preventive role in chronic diseases by safeguarding healthy cells and exuding cytotoxic effects on cancer cell lines (Saldívar‐González et al., 2018). Additionally, the polyphenols in kombucha tea demonstrated the ability to hinder the ROS‐mediated ERK/JNK/p38 pathway and the subsequent cytokines in Wistar rats (Wang et al., 2016). Based on the findings in this study, kombucha tea is able to protect adipose tissues from lipid toxicity and effectively reduce inflammation induced by LSD‐1 dysfunction.

## 5 CONCLUSION

Kombucha tea proved to induce lipase activation, effectively degrading lipids. It attenuated LSD‐1 dysfunction‐induced inflammation in *Drosophila*, which mimics mammalian inflammation. Kombucha tea inhibited the release of proinflammatory cytokines, reduced ROS production, and prevented *p*‐JNK translocation from the cytoplasm to the nuclei in intestinal cells. Our results provide the first strong evidence that kombucha tea could help regulate the JNK signaling pathway by controlling JNK phosphorylation and could additionally provide novel insights into the mechanisms of action in kombucha tea used for the treatment of inflammation and obesity.

## AUTHOR CONTRIBUTIONS

**Duy Binh Tran:** Conceptualization (lead); formal analysis (lead); investigation (lead); methodology (lead); writing – original draft (lead); writing – review and editing (lead). **Nguyen Khoi Nguyen Le:** Methodology (equal). **Minh Tue Duong:** Methodology (equal). **Kamo Yuna:** Methodology (equal). **L. A. Tuan Pham:** Methodology (equal). **Q. C. Thanh Nguyen:** Methodology (equal). **Yingmanee Tragoolpua:** Investigation (equal); methodology (equal). **Thida Kaewkod:** Investigation (equal); methodology (equal); writing – review and editing (equal). **Kaeko Kamei:** Conceptualization (equal); funding acquisition (equal); writing – review and editing (equal).

## FUNDING INFORMATION

There is no funding support.

## CONFLICT OF INTEREST STATEMENT

The authors declare that they have no known competing financial interests or personal relationships that could have appeared to influence the work reported in this paper.

## Supporting information


Figure S1.

## Data Availability

The data that support the findings of this study are available on request from the corresponding author upon reasonable request.
