# Supplementary material for: Drosophila models of the anti‐inflammatory and anti‐obesity mechanisms of kombucha tea produced by Camellia sinensis leaf fermentation
Source: Food Sci Nutr. 2024 May 19;12(8):5722–33. doi: 10.1002/fsn3.4223 (PMC11317715; doi:10.1002/fsn3.4223)
Supplement: Supplementary file 1 — Figure S1. [file FSN3-12-5722-s001.docx]

**Supplementary figures**

**Figure S1. Induction of *Lsd-1* RNAi led to reduced expression of LSD-1.** Midgut of control and *Lsd-1* KD flies were stained with anti-rabbit-LSD-1 antibody, followed by anti-rabbit IgG Alexa Fluor^TM^ 594 antibody (**A** and **B**) and DAPI to visualize DNA (**A′** and **B′**). Both images of DAPI staining and immunostaining results were merged (**A′′** and **B′′**). The images in the figure are representative images for 10 midguts. *Lsd-1* mRNA levels in the midgut of third instar larvae of control and *Lsd-1* KD flies were analyzed using RT-qPCR (**C**, *n*=4). Scale bar, 200 µm. Genotypes: (A**–**A**′′**) +; *Fb*-GAL4/+; UAS-*GFP*-IR, (**B–B′′**) +; *Fb*-GAL4/UAS- *Lsd-1*-IR_170-220_; +
